# Mapping the neural basis of the perception of “flow of time”: interval timing and mental time travel in the human brain

**DOI:** 10.3389/fneur.2026.1960572

**Published:** 2026-10-12

**Authors:** Ryuta Ochi, Mitsuru Kawamura

**Affiliations:** 1 Course of Psychology, Graduate School of Letters, Chuo University, Hachioji, Japan; 2 Division of Neurology, Department of Medicine, Showa Medical University, Shinagawa-ku, Japan

**Keywords:** acquired brain injury, flow of time, interval timing, mental time travel, neurodegenerative diseases

## Abstract

Although neurodegenerative diseases and brain injuries have been reported to impair the perception of “the flow of time,” the underlying neural mechanisms remain uncertain. We examined symptoms related to interval timing and mental time travel, two neuropsychological phenomena associated with the perception of the flow of time that have been relatively well characterized. In addition, we reviewed the current evidence and proposed a potential underlying brain mechanism.

## 1 Introduction

### 1.1 Neuropsychological symptoms related to time

Although philosophical arguments have questioned whether time exists as an objective entity (1), humans perceive and experience time as continuously passing, independently of socially defined (“government”) time, such as “clock-on-the-wall time” (2). This perception of the “flow of time” comprises two principal aspects: the perception of the passage of seconds, minutes, and hours (interval timing) (3), and the perception of the past, present, and future, referred to as mental time travel (or time perspective) (4–6). Two major theoretical accounts have been discussed regarding the neural mechanisms underlying time perception: a single-clock account, which assumes a unitary timing mechanism, and a multiple-clock account, which assumes multiple timing mechanisms that may operate according to task demands and context (7–9). Prior studies have indicated that perception of the flow of time can be impaired following brain injury and in neurodegenerative diseases (10–13). We organized cognitive phenomena related to the flow of time into two categories: impairments in the perception of interval timing and impairments in mental time travel (Table 1).

**Table 1:** Etiologies of impaired perception of flow of time and associated brain regions.

Perception of flow of time	Brain region	Associated disease
Interval timing		
Prospective	Hippocampus Basal ganglia	Alzheimer’s disease Parkinson’s disease
Retrospective	Anterior cingulate gyrus Posterior cingulate gyrus Precuneus Retrosplenial cortex	Hemorrhagic infarction involving the left anterior cerebral artery territory^a^ Tumor involving the left precuneus ^b^
Mental time travel	Anterior cingulate gyrus Posterior cingulate gyrus Precuneus Retrosplenial cortex Hippocampus	Alzheimer’s disease

^a^Futamura et al. (11) and ^b^Skye et al. (27).

## 2 Impairments in the interval timing

Two forms of cognition are associated with the perception of interval timing. One involves situations in which individuals know in advance that they will need to judge the passage of time, whereas the other involves situations in which time is judged retrospectively. These paradigms are referred to as prospective timing and retrospective timing, respectively (3). In prospective timing, individuals know in advance that they will be required to judge a given duration and can direct their attention to the passage of time. This is analogous to estimating when 3 min have elapsed while waiting for instant noodles to be ready after adding boiling water, for example. In contrast, retrospective timing refers to situations in which individuals are asked only after an event to estimate how much time elapsed during an activity in which they were not attending to the passage of time. This is analogous to looking back on a meeting held earlier in the day and estimating how long it lasted, for example. These paradigms involve distinct cognitive processes, with prospective timing relying primarily on attentional functions and retrospective timing relying primarily on memory functions (14, 15). This section summarizes neurological symptoms associated with impairments in the perception of interval timing.

### 2.1 Clock-related time perception symptoms

#### 2.1.1 Prospective interval timing

To investigate impairments in the perception of interval timing, studies have been conducted in patients with Parkinson’s disease (PD). PD is a neurodegenerative disorder characterized by motor symptoms, including tremor and bradykinesia, and is associated with selective loss of dopamine-producing neurons in the nigrostriatal system (16). Although PD is associated with a range of cognitive impairments (17–19), we found that PD has been associated with impairments in the perception of chronological time (20–22). In these studies, we used two tasks to assess the perception of chronological time: the time production task and the time reproduction task. In the time production task, 1 of 11 target durations (0.5, 1, 2, 3, 5, 10, 20, 30, 60, 120, or 300 s) was presented for 3 s at the beginning of each trial. After the target duration disappeared from the screen, participants estimated the interval by pressing a button to indicate its start and end. Participants were instructed to count every 1 s in their heads while estimating the interval. In the time reproduction task, a circle was presented on a laptop screen for 1 of 11 target durations (0.5, 1, 2, 3, 5, 10, 20, 30, 60, 120, or 300 s), and participants were instructed to reproduce the presentation duration by pressing a button after they judged that the same interval had elapsed. The time production task assesses participants’ subjective perception of the passage of time, whereas the time reproduction task requires retention of the target duration and depends, in part, on working memory.

Compared to age-matched controls, PD patients tended to underestimate the passage of time in the time production task by responding before the instructed interval had elapsed (after 20 s instead of 30 s) (20). Conversely, no significant differences were observed between the groups in the time reproduction task. These findings suggest that impaired perception of interval timing in patients with PD may occur independently of working memory demands. Furthermore, PD patients who underestimated the passage of time had lower dopamine transporter binding in the striatum, suggesting that the striatum may represent a neural substrate underlying the perception of time.

We investigated interventions to improve impairments in time perception in PD patients. In a prior study, PD patients repeatedly performed a time production task while observing a clock with a second hand and were instructed to press a button when a specified interval had elapsed (23). Although this training produced an immediate improvement in the perception of interval timing, patients resumed underestimating the passage of time within a few minutes after the training. Because this phenomenon could not be explained by impaired memory function, it was considered to reflect a deficit specific to learning the passage of time. To improve the training method, Honma et al. (22) developed a protocol using a digital stopwatch. Participants estimated intervals of 9 or 11 s without viewing the display, pressed a button to indicate that the target interval had elapsed, and then compared their estimate with the actual elapsed time. After completing this training 10 times daily for 1 month, participants demonstrated improved accuracy of interval estimation at the 1-month follow-up. In addition, improvements in interval estimation accuracy were accompanied by higher accuracy on the Go/No-Go task and longer reaction times. These findings indicate that training to improve interval timing may enhance inhibitory control.

Multiple sclerosis (MS) is a chronic inflammatory demyelinating disease of the central nervous system characterized by focal lesions in the gray and white matter as well as diffuse neurodegenerative changes throughout the brain (24). Impairments in prospective interval timing have recently been reported in patients with MS (25, 26). Echreshavi et al. (25) compared patients with MS and healthy controls using time production, time reproduction, and duration discrimination tasks. Patients with MS overestimated intervals ranging from 100 to 1,000 ms in the time production and duration discrimination tasks. These impairments were not associated with the number of plaques detected via magnetic resonance imaging. In contrast, no significant difference between patients with MS and healthy controls was observed in the time reproduction task. Pourmohammadi et al. (26) examined interval timing in patients with MS using a time reproduction task with intervals ranging from 400 to 1,900 ms. Compared to healthy controls, MS patients showed a stronger tendency to overestimate intervals ≤ 1,100 ms and to underestimate longer intervals. Using a Bayesian observer model, the authors further examined which of three processing stages—sensory measurement, time estimation, or motor reproduction—contributed to this impairment. Their findings suggested that the impairment may arise primarily at the sensory measurement stage.

Studies of interval timing in MS have only recently emerged, and findings across different tasks have not been entirely consistent. Given that MS involves white matter pathology and can disrupt communication across distributed brain regions, interval timing impairments in MS may be understood, at least in part, from the perspective of disrupted neural networks rather than dysfunction of a single timekeeping region. Further investigation of interval timing in MS may therefore provide useful evidence for understanding the distributed neural mechanisms underlying time perception.

#### 2.1.2 Retrospective interval timing

We reported a case of significantly impaired retrospective interval timing following a hemorrhagic stroke, extending from the anterior to the posterior left cingulate gyrus (11). No general cognitive decline was observed; however, mild episodic memory impairment and topographical disorientation were present, and the patient reported that time appeared to pass too quickly. Although the patient was able to count seconds at a normal pace, when asked unexpectedly how much time had elapsed since a test or medical examination, the patient consistently underestimated the elapsed time.

Furthermore, Skye et al. (27) reported multiple cases of impaired retrospective interval timing. Using lesion–symptom mapping, they evaluated 11 patients with brain injury who had impaired temporal orientation despite no apparent memory impairment and identified the left posterior cingulate cortex and right precuneus as the common lesion site. Temporal orientation refers to the cognitive function that aligns an individual’s subjective perception of time with clock time (28–30). In addition, two patients with brain tumors involving the medial aspect of the left parietal lobe, including this region, reported that “time did not run,” indicating that they perceived the passage of time as longer than the actual elapsed time (27). However, it should be noted that the reports of patients experiencing that “time did not run” are limited to individual case observations (11, 27), and this specific phenomenon has not yet been examined using group-level lesion–symptom mapping.

### 2.2 Differences in the neural bases of prospective and retrospective interval timing

As discussed above, prior studies have suggested that impairments in prospective interval timing are associated with striatal dysfunction. In contrast, retrospective interval timing is more susceptible to impairment following damage to the medial aspect of the left parietal lobe, particularly the precuneus. The basal ganglia, including the striatum, and the prefrontal cortex have been reported to form a functional network that plays an important role in inhibitory control (31, 32).

In contrast, the precuneus is recognized as a hub of the default mode network, a resting-state network (33). The default mode network has been shown to be activated during memory processing, particularly during autobiographical memory retrieval (34). Psychological studies suggest that prospective interval timing relies primarily on attentional functions, whereas retrospective interval timing depends primarily on memory functions (15). The distinct patterns of cognitive impairment and regional brain dysfunction observed in neurological disorders are consistent with these psychological findings, suggesting that prospective and retrospective interval timing are supported by partially distinct neural mechanisms.

Regarding the neural mechanisms underlying time perception, single-clock and multiple-clock accounts have been discussed (7–9). More recently, a computational modeling study suggested that time reproduction and time discrimination may rely on different timing mechanisms, providing support for a multiple-clock account (35). These findings suggest that, even within interval timing, different timing mechanisms may be recruited depending on task demands. From a neuropsychological perspective, the dissociation between the neural substrates associated with prospective and retrospective interval timing reviewed here may also be consistent with a multiple-clock account, in which time perception depends on multiple, task-dependent timing mechanisms rather than a single centralized clock. Furthermore, considering the findings in MS, interval timing may depend not only on specific brain regions but also on interactions among distributed brain regions mediated by white-matter pathways.

## 3 Time symptoms as a disorder of mental time travel

Mental time travel refers to the ability to relive past autobiographical experiences and simulate future events. It is supported by coordinated activity across multiple brain regions rather than by a single neural structure. Although mental time travel has been studied primarily as a memory function, this section is focused on cognitive representations of concepts of the “past,” “present,” and “future” within the framework of mental time travel.

Impairments in mental time travel are well documented in patients with dementia. In our study, we conducted interviews with patients with Alzheimer’s disease (AD) to assess their perceptions of the “past,” “present,” and “future” (13). Patients with AD described their experiences using expressions, such as “the past comes up” and “going back and forth between the past and the present.” In contrast, they reported that they “cannot imagine the future,” suggesting difficulty in constructing future events. Previous studies using autobiographical memory tasks have shown that AD patients exhibit impairments in memory recall (36). However, our findings suggest that representations of the “past” and “present” may emerge as mental images that appear to merge, rather than remain temporally distinct (13).

We conducted a study using a temporal judgment task in AD patients and those with mild cognitive impairment (MCI) (12). In this task, participants judged the temporal location of events. For instance, they determined whether a statement, such as, “I ate curry rice yesterday,” referred to the past, present, or future. A functional magnetic resonance imaging study in healthy adults found that the precuneus is activated during judgments involving the present tense (37). When we administered this task to AD and MCI patients, AD patients demonstrated impairments in judgments involving the “past,” “present,” and “future” (12). In contrast, MCI patients were generally able to distinguish between the past, present, and future but had difficulty making finer temporal distinctions. For example, they had difficulty judging that “last month” was temporally more distant than “yesterday” (12). Furthermore, we evaluated the association between performance on the temporal judgment task and resting-state cerebral blood flow measured by single-photon emission computed tomography. Poorer task performance was associated with reduced cerebral blood flow in the bilateral pericallosal regions, including the precuneus and cingulate cortex (12).

## 4 Discussion

### 4.1 Relationship between interval timing and mental time travel

McTaggart (1) distinguished two ways of ordering events in time: the A-series, in which events are characterized as past, present, or future, and the B-series, in which events are ordered as earlier than, simultaneous with, or later than other events. Although it remains to be determined whether interval timing and mental time travel, as discussed in this mini-review, correspond directly to these philosophical concepts, McTaggart’s distinction provides a conceptual framework for considering different aspects of temporal cognition.

Both interval timing and mental time travel may represent aspects of what Gooddy (2) termed “personal time,” a concept of time based on multitudinous physiological clock systems rather than government time. The relationship between interval timing and mental time travel remains unclear. However, from a theoretical perspective, John W. Brown proposed the microgenesis hypothesis (38, 39). According to this hypothesis, the “phenomenal now,” which corresponds to the perceived present, emerges from a sense of temporal distance generated by the decay of time represented on the millisecond timescale, referred to as the “absolute now” (38). Furthermore, the hypothesis proposes that temporal representations of the past and future are constructed through the process of generating the present (39).

### 4.2 The neural basis of the perception of “the flow of time”

Consistent with the multiple-clock account introduced above, the neuropsychological evidence reviewed here suggests that the perception of the flow of time may arise from interactions among multiple brain regions rather than from a single centralized timekeeping mechanism. Thus, these findings are broadly consistent with a multiple-clock or distributed-systems account of time cognition. Figure illustrates our proposed neural network model of the perception of the flow of time. The arrows represent hypothesized network relationships between brain regions based on previously reported functional connectivity (7, 37, 40, 41). Previous studies have suggested that the precuneus and adjacent posterior midline structures provide a neural anchor for the subjective experience of temporal presentness (37). We propose a model of the brain regions implicated in the perception of the flow of time based on Kawamura et al. (42). The medial parietal cortex, including the precuneus, posterior cingulate cortex, and retrosplenial cortex, plays a central role in this model. As discussed earlier, functional neuroimaging studies have demonstrated that bilateral activation of the precuneus is strongly associated with the perception of the present (37). These regions are interconnected within the default mode network, which is related to mental time travel (41). In addition, interval timing has been linked to the basal ganglia and prefrontal cortex (7, 40). These findings suggest that the perception of the flow of time emerges from coordinated processing across these interconnected neural networks (Figure 1).

**Figure 1:**
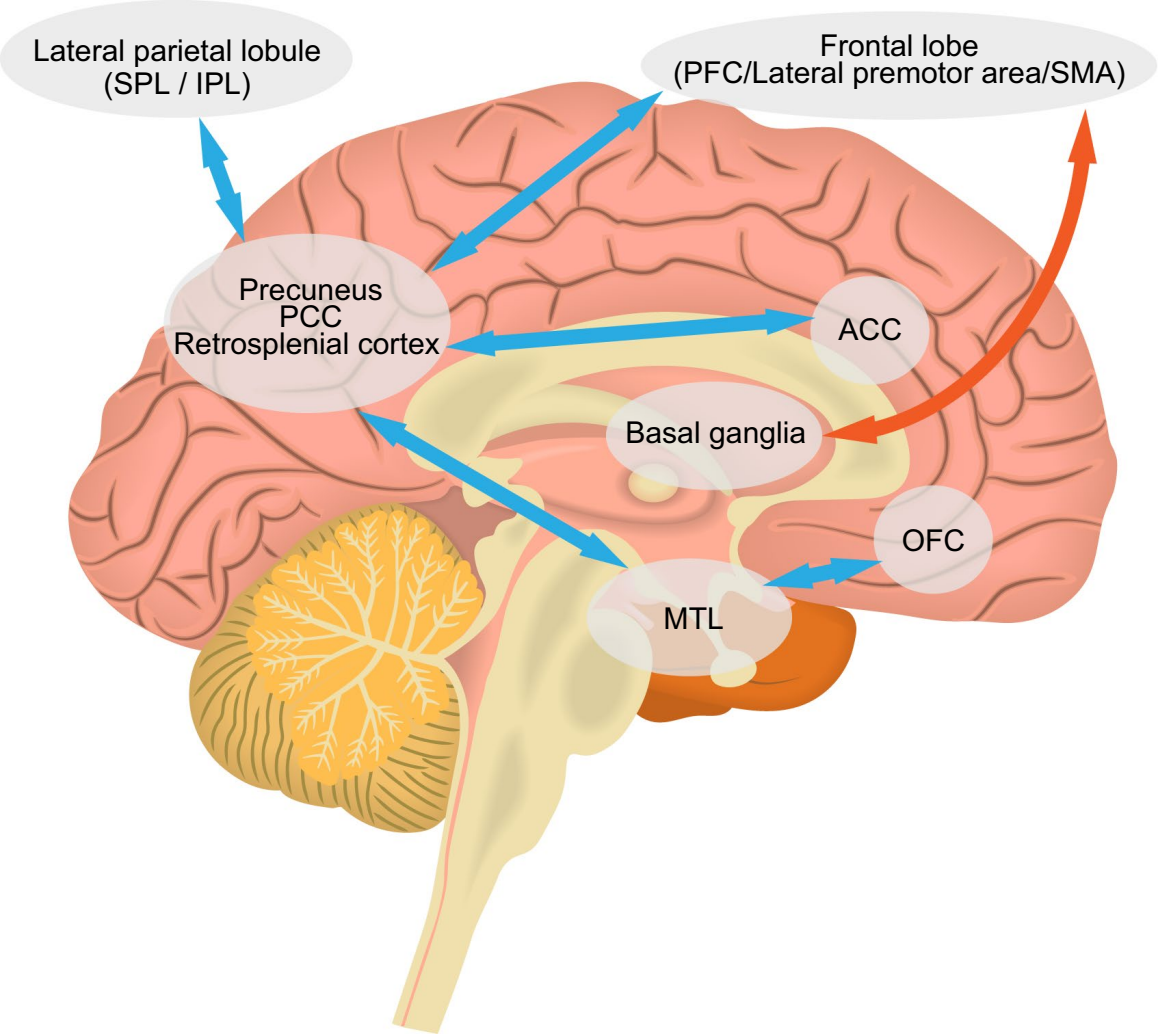
Hypothetical neural network underlying the perception of the “flow of time.” Caption: ACC, anterior cingulate cortex; IPL, inferior parietal lobule; MTL, medial temporal lobe; OFC, orbitofrontal cortex; PCC, posterior cingulate cortex; PFC, prefrontal cortex; SMA, supplementary motor area; SPL, superior parietal lobule. Arrows represent hypothesized network relationships between brain regions based on previously reported functional connectivity. Orange arrows represent the network associated with prospective interval timing, whereas blue arrows represent the network associated with mental time travel. The conceptual framework was adapted from (42), with permission from the original publisher. The brain illustration was adapted from ‘Mid-sagittal plane of the brain’ by the DataBase Center for Life Science (DBCLS), DOI: 10.7875/togopic.2021.023, licensed under CC BY 4.0.

### 4.3 Limitations and future directions

Several limitations of this mini-review should be acknowledged. First, although philosophical inquiry into the nature of time has a long history, neuropsychological research on the perception of the flow of time is a relatively recent field. A substantial proportion of the studies reviewed in this mini-review were conducted by our research group; evidence from other institutions and research groups remains limited. Thus, the evidence base reviewed here may be subject to bias arising from the concentration of studies within a single research group. Furthermore, much of the available evidence is based on case reports or studies with relatively small samples, limiting the generalizability of the findings. Future large-scale multicenter studies and quantitative meta-analyses are therefore necessary to evaluate the validity of our proposed model. Second, the neuroimaging study discussed in this review (12) examined correlations between functional neuroimaging findings and behavioral data and therefore does not establish a causal relationship between brain dysfunction and impaired perception of the flow of time. Future longitudinal studies, lesion–symptom mapping, and computational modeling studies are needed to better characterize the mechanisms underlying impaired perception of the flow of time. Third, although we propose a neural network model of the perception of the flow of time, whether this model is also applicable to disorders predominantly involving white matter pathology, such as MS, has not yet been established. Future studies of patients with MS and other disorders involving white matter lesions, such as leukoencephalopathy, are needed to test and further refine the proposed network model.
